# Minimally Invasive Decompression for Chiari I Malformation: A Systematic Review and Meta-Analysis

**DOI:** 10.7759/cureus.89016

**Published:** 2025-07-29

**Authors:** Patrick Pema, Daniel Monahan, Shiv Patel, Nitesh V Patel, Kevin Zhao

**Affiliations:** 1 Neurosurgery, Hackensack Meridian Jersey Shore University Medical Center, Neptune, USA; 2 Medicine, Hackensack Meridian School of Medicine, Nutley, USA

**Keywords:** cerebrospinal fluid, chiari i malformation, endoscopic, minimally invasive, pseudomeningocele

## Abstract

Chiari I malformation (CIM) is a structural defect in the cerebellum, which is characterized by herniation of the cerebellar tonsils into the foramen magnum. While open foramen magnum decompression is traditional, some surgeons practice a minimally invasive technique. Our paper intends to further elucidate the differences in outcomes between the traditional and minimally invasive foramen magnum decompression (MID). Article selection was from PubMed. Included studies had symptomatic patient populations requiring surgical intervention via MID, and whose complications were explicitly reported and described. A total of 200 patients were reported by 10 studies. Surgical incision area ranged from 2.5 cm² to 12 cm². Meta-analysis revealed that operative time was highly heterogeneous (I² = 99%). Neurological testing included visual analogue scale (VAS), Karnofsky performance scale (KPS), modified Japanese Orthopaedic Association score (mJOA), and Chicago Chiari outcome scale (CCOS). The average rate of patient syringomyelia improvement was 84%. Meta-analysis of complication rates showed a moderate heterogeneity (I² = 61%), with common and random effect models yielding proportions of 0.16 and 0.12, respectively. The most and least common complications were dural tear (eight occurrences) and superficial wound infection (one occurrence). Averages of neurological testing scales confirmed improvement in function. Syringomyelia improvement rates demonstrated that MID is effective. Complication rates were found to be favorable and superior to those of open surgery. These results demonstrate that this technique is effective for the treatment of symptomatic CIM and that MID is at least as efficacious as open surgery.

## Introduction and background

Chiari I malformation (CIM) is characterized by herniation of the cerebellar tonsils into the foramen magnum; this condition can be either genetic or acquired in origin. Such herniation results in the disrupted flow of cerebrospinal fluid (CSF) and can manifest with a variety of symptoms. As such, CIM can result in hydrocephalus and/or syringomyelia and is associated with spinal deformities such as scoliosis and kyphosis [1]. Syringomyelia, a condition in which a fluid-filled cyst forms in the spinal cord and compresses adjacent neuronal tracts, is highly associated with CIM and is a common indication for surgical correction [2]. Common symptoms include headache, neck pain, dizziness, trouble swallowing, numbness or tingling of extremities, and subtle weakness.

Patients with CIM are divided into two categories: those with and without a syrinx. Patients with a syrinx are often treated with surgical decompression [2]. Surgical intervention for patients without a syrinx is reserved until the development of lifestyle-limiting headaches or objective neurological abnormalities. For patients with coexisting hydrocephalus, CSF diversion should be taken into consideration.

Suboccipital decompression of the foramen magnum through open surgery is the mainstay of surgical treatments for CIM. Removal of the first, second, or even third vertebral lamina may be indicated depending on the extent of the cerebellar descent. The patient is positioned prone, and an incision is made to expose the foramen magnum and posterior arch of C1. Bone is removed from the subocciput and C1 posterior arch to relieve compression and restore CSF flow with or without duraplasty [1]. Intraoperative ultrasonography is performed to assess tonsillar movement and CSF flow to aid in the decision to perform C1 laminectomy or duraplasty. This procedure aims to restore the normal flow of CSF and decompress any compression at the cervicomedullary junction.

Minimally invasive decompression (MID) for CIM was first described in the early 2000s, and different variations are now practiced by many surgeons around the world. Such minimally invasive techniques for CIM can be either through a small incision and rely on the angling of self-retaining or tubular retractors or the surgeries can be done through an endoscope.

MID through small openings with tubular or small self-retaining retractors can be used to achieve a large bone opening and graft area by tilting the retractors to gain the angles needed. An endoscopic approach can be done generally with two practitioners, with one holding the endoscope to gain the angles needed to reach under the skin. Generally, the patient is positioned prone on the operating room table with the head elevated and fixed. A small midline incision is made at the level of the foramen magnum. A small self-retaining retractor or a tubular retractor is inserted. By tilting the retractor, angles are gained to dissect soft tissues off the bone. Craniotomy and C1 laminectomy are done with standard techniques. The fibrous restrictive band of ligament at the cervicomedullary junction is cut. Intraoperative ultrasound is used to assess adequate CSF flow. If adequate CSF flow is noted, then a satisfactory decompression has been completed. If inadequate CSF is noted, then the dura is opened, and a duraplasty is performed. Depending on the severity of the tonsillar herniation, some physicians may conduct either a unilateral or bilateral cerebellar tonsillectomy.

For the traditional large opening approach, the most common postoperative complications are pseudomeningocele, CSF leak, and bacterial meningitis [3]. Advancements in the treatment of CIM have focused on improving the occurrence of complications and reducing factors such as hospital stay and blood loss. Traditional open techniques to treat CIM have proven to be safe and effective; however, MID techniques have been shown to achieve comparable results [4], with the benefits of minimal invasiveness, early recovery, reduced hospital stay, and less expense. Some surgeons have expressed concerns regarding added surgical time with endoscopic or tubular techniques compared to the traditional approach, as well as limited bony removal and a reliance on tonsillar shrinkage to achieve favorable decompression.

MID provides promising benefits over traditional open surgery. Open procedures involve a larger, 5-10 cm incision, wider muscular dissection, and higher intraoperative blood loss [4,5]. MID involves a 2-3 cm incision, limited musclar dissection, results in less blood loss than the open approach on average [4,5], and may be performed through an endoscope. In addition, many MID procedures often take a 'dural-sparing' approach, where the opening of the dura is either limited or avoided entirely; this technique lowers the risk of infection [3,5].

As of our review, no literature exists to comprehensively describe variations in technique, outcomes, and characteristics of MID procedures practiced by surgeons across the world. Our paper intends to further describe the efficacy, safety, and efficiency of minimally invasive techniques to treat CIM while highlighting how those characteristics compare to traditional, open techniques. By creating a comprehensive review, this study may inform surgeons and patients of the effectiveness and safety profile of minimally invasive foramen magnum decompression to treat CIM.

## Review

Methods

**Figure 1:**
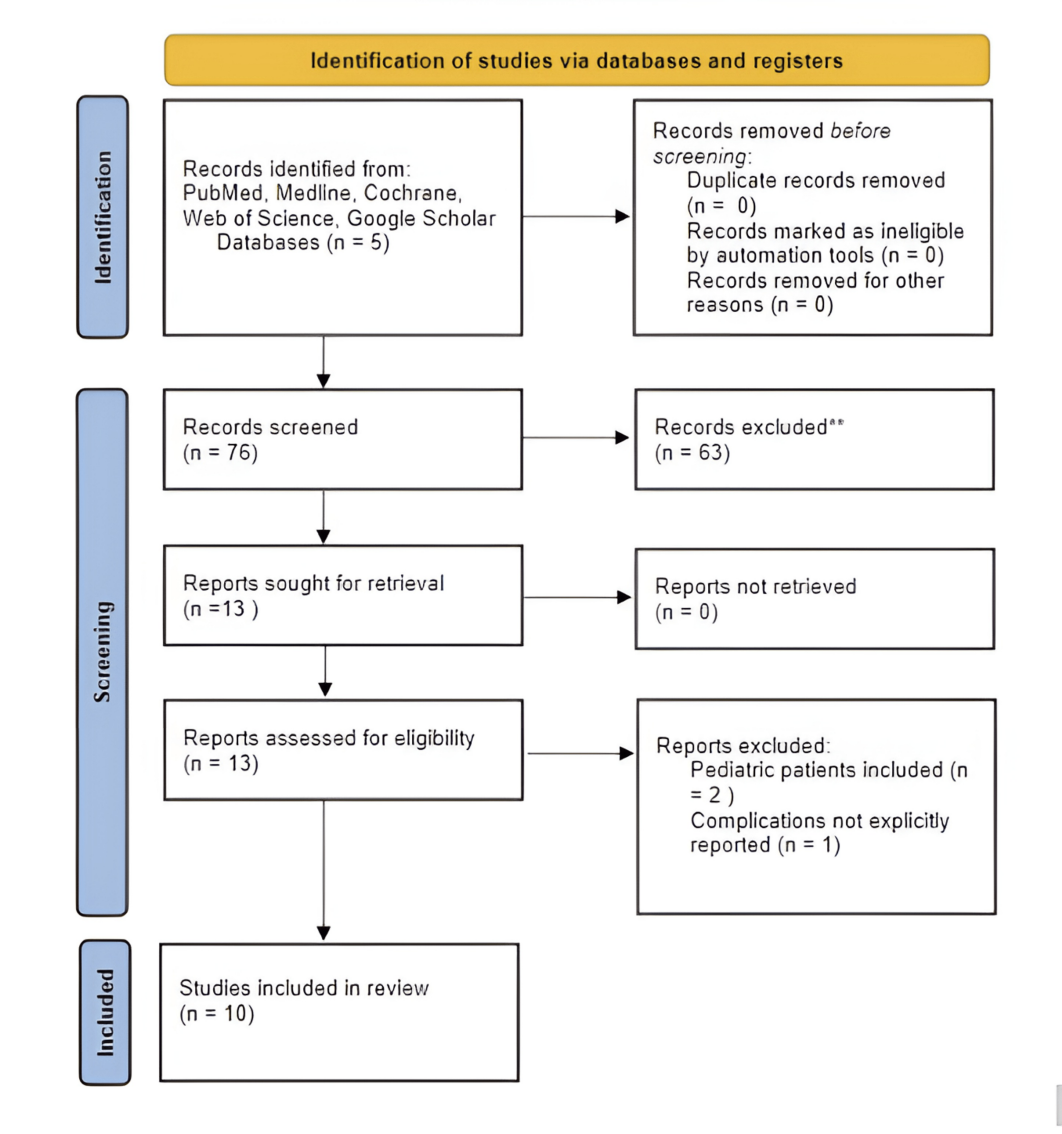
A PRISMA flowchart describing the review process. PRISMA: Preferred Reporting Items for Systematic Reviews and Meta-Analyses

An Institutional Review Board approval was not necessary for this study because it did not meet the criteria for human subject research. This systematic review was conducted using the guidelines established by the Preferred Reporting Items for Systematic Reviews and Meta-Analyses (PRISMA). This review was not registered in Prospero. Article selection was from PubMed database on July 24, 2024. A PRISMA diagram describing the selection process is depicted in Figure 1.

Eligibility Criteria

Articles were not excluded due to their date of publication. Inclusion criteria were as follows: (1) patient population who were experiencing symptoms and required surgical intervention due to CIM, (2) studies that included a patient group who underwent minimally invasive (neuroendoscopic) technique for foramen magnum decompression, and (3) adult (>18 years of age) patient population. Study exclusion criteria were as follows: (1) pediatric patients and (2) post-surgical complications not reported or explicitly described.

Data Collection, Search Strategy, and Information Sources

The search process consisted of two stages. The first stage was a literature review, which was conducted via a thorough search of medical literature databases (PubMed, Medline, Cochrane Library, Web of Science, and Google Scholar). The exact boolean strings used for search are described here: ("Chiari I malformation" (MeSH Terms) OR "Chiari I malformation" (Title/Abstract) OR "Chiari malformation type 1" (Title/Abstract) OR "Arnold Chiari" (Title/Abstract)) AND ("minimally invasive" (Title/Abstract) OR "endoscopic" (Title/Abstract) OR "tubular retractor" (Title/Abstract) OR "neuroendoscopy" (Title/Abstract)) AND ("foramen magnum decompression" (Title/Abstract) OR "suboccipital decompression" (Title/Abstract)). The search criteria yielded 76 results. These articles and their abstracts were reviewed by two authors (PP and SP) who pared the number of relevant articles down to 13. The second stage, inclusion and exclusion, of which the criteria are described above, was performed by one author (PP). This stage yielded 10 relevant articles [5-14], whose inclusion in the study was confirmed by the principal investigator (KZ). 

Risk-of-Bias Assessment

The quality of the included studies was assessed using the MINORS (methodological index for non-randomized studies) quality assessment tool [15], a validated instrument specifically designed to evaluate the methodological quality of non-randomized studies. The results of the quality assessment are displayed in Table 1. Each study received a total score out of 16 (non-comparative) or 24 (comparative). Scores were used to guide interpretation of results, with studies of lower methodological quality considered more cautiously in the analysis.

**Table 1:** Quality assessment of each study as per the MINORS (methodological index for non-randomized studies) quality assessment tool. All studies included achieved at least moderate methodological quality.

Year	Lead Author	Study type	Quality Assessment Tool	Score	Description
2018	Teo [5]	Retrospective cohort study with a comparative analysis.	MINORS Assessment	14/24	Moderate methodological quality
2024	Günerhan [6]	Prospective observational study	MINORS Assessment (Non-comparative version)	11/16	Moderate methodological quality
2018	Kotil [8]	Retrospective cohort study	MINORS Assessment (Non-comparative version)	9/16	Moderate methodological quality
2023	Mostofi [9]	Retrospective comparative study	MINORS Assessment	16/24	Moderate methodological quality
2019	Caffo [10]	Case series	MINORS Assessment (Non-comparative version)	10/16	Moderate methodological quality
2024	Günerhan [7]	Retrospective case-control study	MINORS Assessment	20/24	High methodological quality
2024	Costa [11]	Case series	MINORS Assessment (Non-comparative version)	11/16	Moderate methodological quality
2019	Zagzoog [12]	Case series	MINORS Assessment (Non-comparative version)	11/16	Moderate methodological quality
2017	Pakzaban [13]	Case series	MINORS Assessment (Non-comparative version)	11/16	Moderate methodological quality
2018	Ratre [14]	Prospective cohort study	MINORS Assessment (Non-comparative version)	10/16	Moderate methodological quality

The meta-analysis and statistical computations were performed using R (version 4.3.1; R Foundation for Statistical Computing, Vienna, Austria) with the meta and metafor packages for pooling effect sizes, generating forest plots, and calculating heterogeneity statistics.

Results

Study Characteristics

The study characteristics are described in Table 2. The year of publication ranged from 2017 to 2024. Three took place in North America, five were conducted in Europe, and two took place in Asia. Four were case series, three were cohort studies, two were case-control studies, and there was a single observational study. Three studies included an open surgery group for comparison; the remaining seven used only a minimally invasive technique. The total number of patients on which operations were performed was 264; 64 underwent the standard open technique, and 200 were operated on using a minimally invasive technique. All but one author [12] described the post-operative follow-up time, which ranged from six weeks [9] to 88 months [8].

**Table 2:** Study characteristics. CIM: Chiari I malformation; MID: Minimally invasive decompression

Year	Lead Author	Country	Study type	N	Participant groups	Follow-up period
2018	Teo [5]	Singapore	Retrospective cohort study with a comparative analysis	14	Open (5) and MID (9)	37.2 months (range: 13-70 months)
2024	Günerhan [6]	Denmark	Prospective observational study	22	MID	6-24 months
2018	Kotil [8]	Turkey	Retrospective cohort study	61	MID - Chiari I (53 patients) and Chiari 1.5 (8 patients)	Mean of 5.5 years (range: 44-88 months)
2023	Mostofi [9]	France	Retrospective comparative study	12	Open (6) and MID (6)	6 weeks, 3 months, 6 months, 1 year
2019	Caffo [10]	Italy	Case series	26	MID	27.5 months (range: 5-72 months)
2024	Günerhan [7]	Denmark	Retrospective case-control study	76	MID (23) and Open (53)	3, 6, 12, 24 months
2024	Costa [11]	USA	Case series	10	MID	6 months
2019	Zagzoog [12]	Canada	Case series	22	MID	None given
2017	Pakzaban [13]	USA	Case series	6	MID	13.2 months (range: 9-18)
2018	Ratre [14]	India	Prospective cohort study	15	MID	9-21 months

Surgical Incision and Laminectomy Area

The operation characteristics are detailed in Table 3. In every study, all patients underwent a craniectomy and C1 laminectomy with posterior fossa decompression. Seven out of 10 studies measured and described the area of bony decompression. The smallest area was listed by Mostofi et al. [9], whose decompression area was 2.5 x 2.5 cm (6.25 cm^2^). Four authors (Kotil et al. [8], Caffo et al. [10], Costa et al. [11], and Zagzoog et al. [12]) reported a 3 x 3 cm (9 cm^2^). Günerhan et al. [6,7] utilized a range of areas, from 9-12 cm^2^, in two separate studies on both of which Günerhan was the lead author.

**Table 3:** Operative characteristics.

Lead Author	Mean operative time (min)	Minimally Invasive Technique used	Incisions	Decompression area
Teo [5]	140.1	Endoscopy guided craniectomy and C1 laminectomy	3 cm midline incision	None described
Günerhan [6]	105	Neuroendoscopic suboccipital craniectomy and C1 laminectomy	Single skin incision from 0.5 cm inferior to the posterior tuberculum of the atlas to 1.5-2 cm toward the superior midline intersection	9-12 cm^2^
Kotil [8]	75	Mini-incision foramen magnum decompression, C1 laminectomy, and C2 medial inner side drill	A single 1.5 cm incision	3 cm x 3 cm
Mostofi [9]	64	Paramedian suboccipital hemicraniectomy and hemi- laminectomy	2 paramedian 3 cm incisions, each 2 cm from the midline	2.5 cm x 2.5 cm
Caffo [10]	None listed	Mini-incision suboccipital craniectomy with C1 laminectomy and Y-shaped dural incision	1 cm above the inion to the spinous process of C2	3 cm x 3 cm
Günerhan [7]	101.82	Neuroendoscopic suboccipital craniectomy and C1 laminectomy	Single skin incision from 0.5 cm inferior to the posterior tuberculum of the atlas to 1.5 to 2 cm toward the superior midline intersection	9-12 cm^2^
Costa [11]	59	Suboccipital craniectomy, C1 laminectomy with tubular retractors and Y-shaped dural incision	3-4 cm	3 cm x 3 cm
Zagzoog [12]	None listed	Suboccipital craniectomy, C1 laminectomy using METRx tubular Retractor and operative microscope	"small midline incision"	3 cm x 3 cm
Pakzaban [13]	114	Limited suboccipital craniectomy and C1 laminectomy with partial C2 laminectomy if necessary using speculum retractors	3-4 cm	None described
Ratre [14]	130	Endoscopy guided craniectomy and C1 laminectomy	2.5-3 cm	None described
Average	98.615			

Mean Operative Time

In a prospective study, Teo et al. [5] found a reduced mean operative time of 140.1 (20.4) for minimally invasive surgery. Günerhan et al. [6] found a mean operative time of 105 minutes for minimally invasive surgery assisted by neuroendoscopy for CIM. Kotil et al. [8] recorded a mean operative time of 75 minutes for both type I and 1.5 malformations; Chiari 1.5 malformations were not included in our study. Mostofi et al. [9] recorded a mean operative time of 64 minutes. In a comparative analysis on open surgery or neuroendoscopic foramen magnum decompression on CIM, Günerhan et al. [6] recorded a mean operative time of 101.82 ± 20.39 minutes. In a surgical technique and preliminary case series studying Chiari I decompression without durotomy, Costa et al. [11] recorded a mean operative time of 59 (59-71) minutes. In a surgical technique paper exploring mini-open decompression of Chiari type I malformation, Pakzaban et al. [13] recorded a mean operative time of 114 minutes (range: 94-153 minutes). In a surgical technique paper exploring endoscopic management of CIM with or without preoperative syringomyelia, Ratre et al. [14] recorded a mean operating time of 130 minutes (110-190 minutes). The average mean operative time of all studies was 98.615 minutes, with a standard deviation of 30.0451.

**Figure 2:**
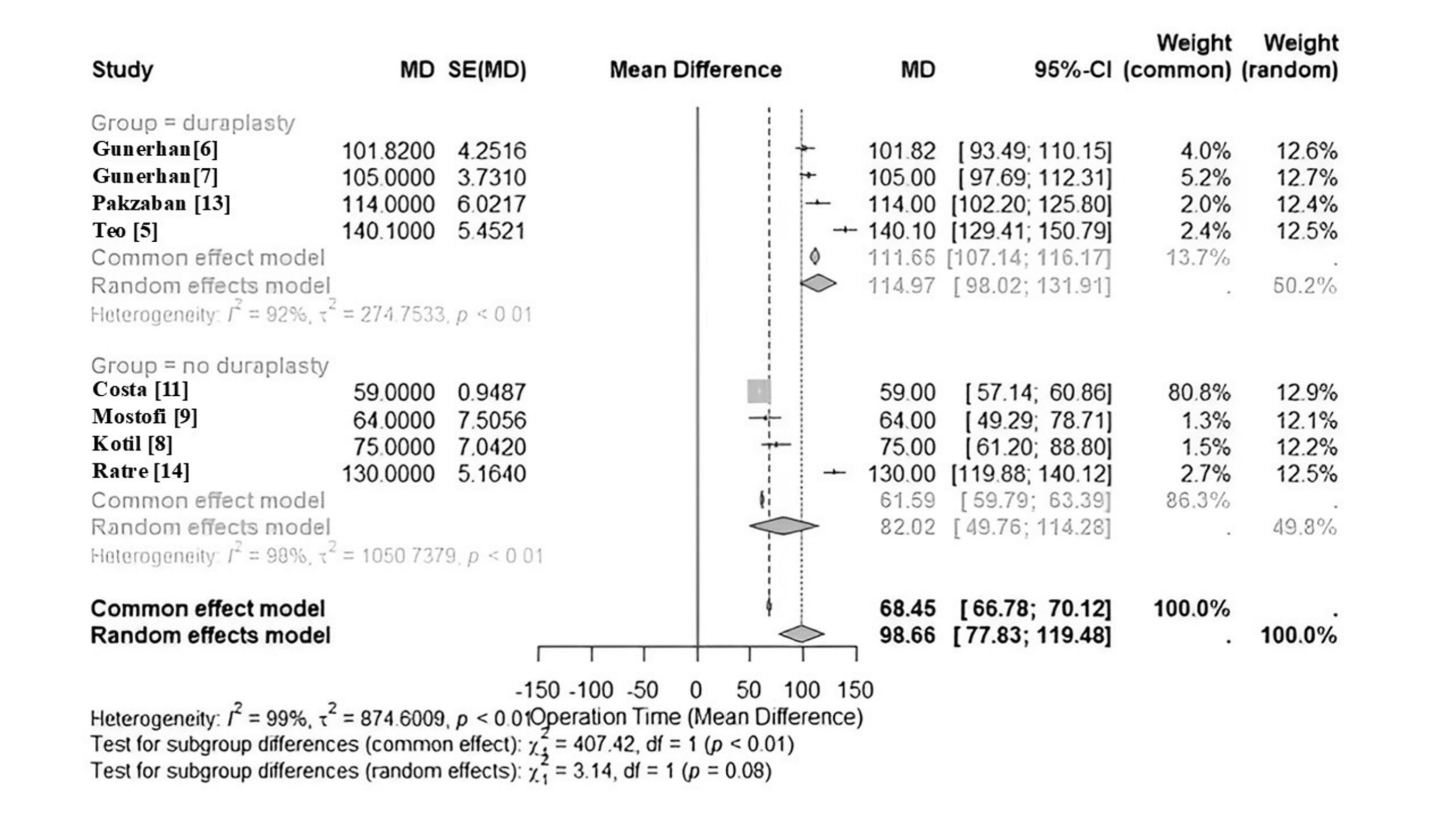
Forest plot displaying two groups: studies where patients underwent duraplasty and studies where duraplasty was not performed. In the duraplasty group, the mean operative times using both a common and random effects model were 111.65 and 114.97 min, respectively. In the non-duraplasty group, the mean operating time using common and random effects models were 68.45 and 98.66, respectively. Both groups demonstrated high heterogeneity with I^2^ values of 92% (duraplasty) and 98% (non-duraplasty).

Eight out of 10 [5-9,11,13,14] studies reported the mean operation duration. Four of those studies involved patients who had the possibility to undergo duraplasty during the procedure, a decision which was made by the surgeon intraoperatively. Four studies included only patients who did not undergo duraplasty [8,9,11,14]. A forest plot describing both groups is depicted in Figure 2.

Outcomes measures

Modified Japanese Orthopaedic Association Score (mJOA)

A measure of post-operative success is the comparison of the mJOA score pre- and post-operatively [16]. In a magnum decompression vs open surgery for type 1 Chiari malformations, Günerhan et al. [6] reported pre- and post-operative MJOA scores of 14.5 ± 1.6/15.6 ± 1.0, respectively.

Visual Analogue Scale (VAS)

**Figure 3:**
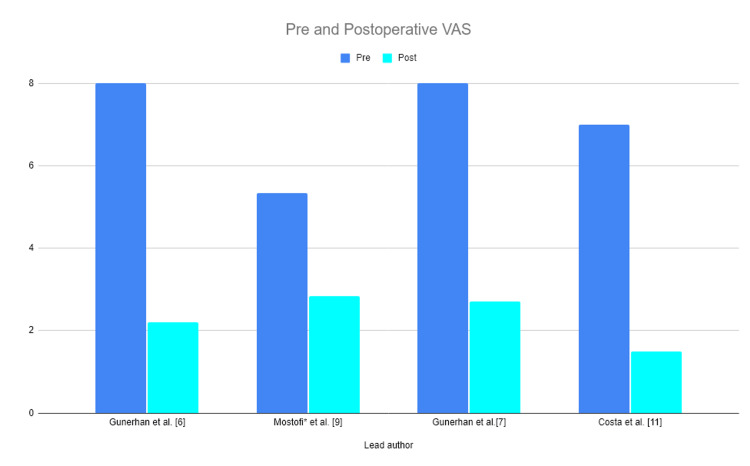
Pre- and post-operative visual analogue scale (VAS) scores. The average VAS score improvement was 4.78. *Mostofi et al. [9] assessed the VAS score on day 0 after operation instead of pre-operatively. The average improvement in the VAS scores was 4.78.

In the context of Chiari malformation correction, and for that matter, other surgical techniques as well, a VAS score is often used to measure success. Pre- and post-operative VAS scores are depicted in Figure 3. In a neuroendoscopy-assisted study, Günerhan et al. [6] reported pre- and post-operative VAS scores of 8.0 ± 1.1 and 2.2 ± 1.1, respectively. Mostofi et al. [9] reported VAS scores of 2.83 one month post-operatively for the minimally invasive surgery. In a comparative analysis assessing neuroendoscopic foramen magnum decompression vs open surgery for type 1 Chiari malformations, Günerhan et al. [7] reported pre- and post-operative VAS scores of 8.0 ± 1.1 and 2.7 ± 1.6 for the minimally invasive surgeries, respectively. In a surgical technique and preliminary case series studying Chiari type 1 decompression without durotomy, Costa et al. [11] reported a VAS score of 1.5 for minimally invasive surgeries. The average pre- and post-operative VAS scores for minimally invasive surgery were 7.08 and 2.31, respectively.

Other Metrics

Another measure of patient improvement is the comparison of the Karnofsky performance scale (KPS) pre- and post-operatively [17]. Two studies chose to measure patient outcomes using the KPS. In a neuroendoscopy-assisted study, Günerhan et al. [6], the authors report pre- and post-operative KPS scores of 81.8 ± 10.5/86.8 ± 6.5, respectively. Ratre et al. [14] reported the average pre- and post-operative KPS scores of 78 and 93, respectively. In addition to the KPS, the Chicago Chiari outcome scale (CCOS) is often used to measure surgical outcomes by assigning scores of 1-4 to post-operative pain, non-pain symptoms, functionality, and complications [18]. Two studies chose to measure patient outcomes using the CCOS. In the same neuroendoscopy-assisted study, Günerhan et al. [6] recorded a CCOS rating of 20 (87%) for the minimally invasive surgery. In a surgical technique paper, Pakzaban et al. [13] reported a CCOS rating of 15.5. 

Syringomyelia Improvement

**Figure 4:**
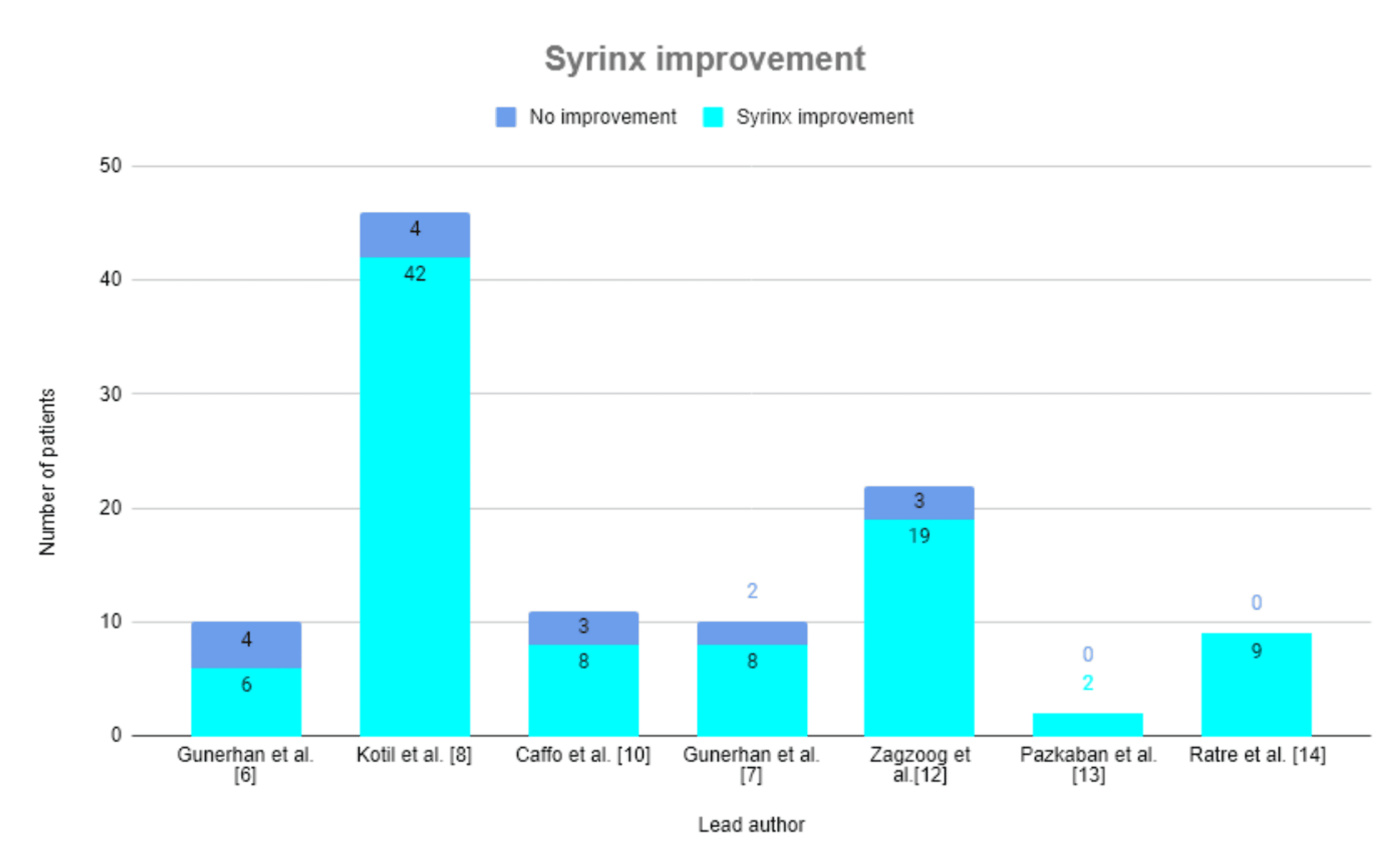
Syrinx improvement. Seven studies reported the proportion of patients whose syrinx improved. Two studies demonstrated syringomyelia improvement in all patients [13,14].

Improvement in the syrinx volume is described in Figure 4. Five studies included patients who had syringomyelia prior to operation. All studies reported an improvement (reduction) in syrinx volume of at least 60%, and the average rate of patient syringomyelia improvement was 84%. The study by Kotil et al. [8] reported the greatest improvement, in which 92% of patients saw a reduction in the syrinx volume. Pazkaban et al. [13] saw resolution of both cases of syringomyelia. Ratre et al. [14] described an improvement in the syrinx volume of at least 50% in all patients.

Complications 

**Figure 5:**
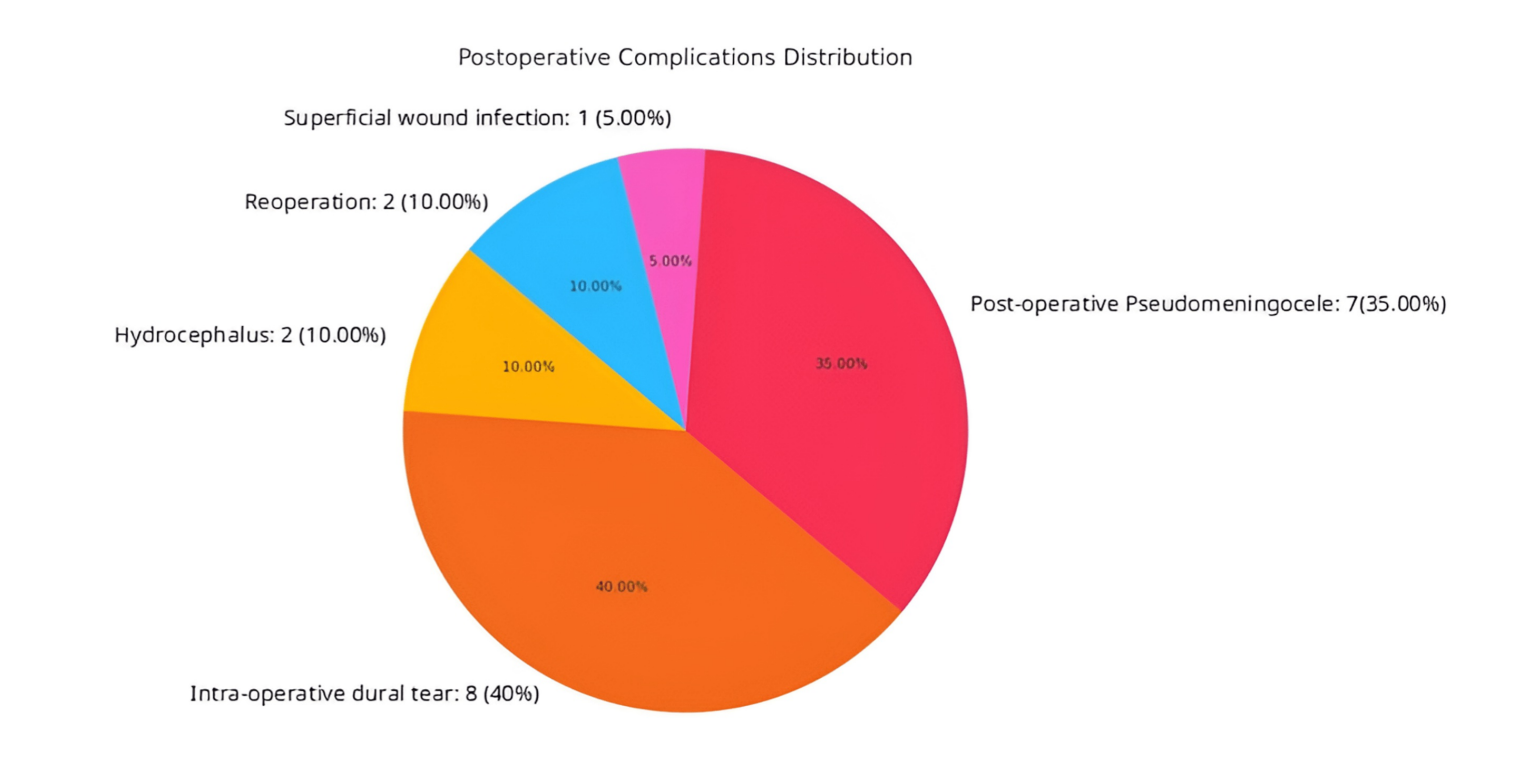
Pie chart showing the proportion of each post-operative complication. The rates of each type of complication are as follows: hydrocephalus (two) [5,13], intra-operative dural tear (eight) [6,7], post-operative pseudomeningocele/CSF leak (seven) [5,6,10,13], superficial wound infection (one) [8] and reoperation (two) [6,7].

A pie chart describing post-operative complications is displayed in Figure 5. Among all minimally invasive surgery groups across all studies, intra- or post-operative complications were uncommon. The average complication rate of all studies was 11%. An exception must be noted: Teo et al. [5] described operations on five patients, of which two developed complications. When removing this specific study and its small number of patients from the average, the mean complication rate drops to 7.5%. A total of 200 patients were operated on using the minimally invasive technique. The total number of patients with specific complications are as follows: two developed post-operative hydrocephalus [5,13], eight experienced intra-operative dural tear (e.g., [6,7]), seven developed post-operative pseudomeningocele/CSF leak (e.g., [5,6,10,13]), one experienced a superficial wound infection [8], and two were required to undergo reoperation due to inadequate decompression [6,7]. One patient out of seven with pseudomeningocele/CSF leak developed a CSF leak, which required reoperation [5]. Notably, three out of seven cases of pseudomeningocele/CSF leak came from Teo et al.'s [5] patient population.

**Figure 6:**
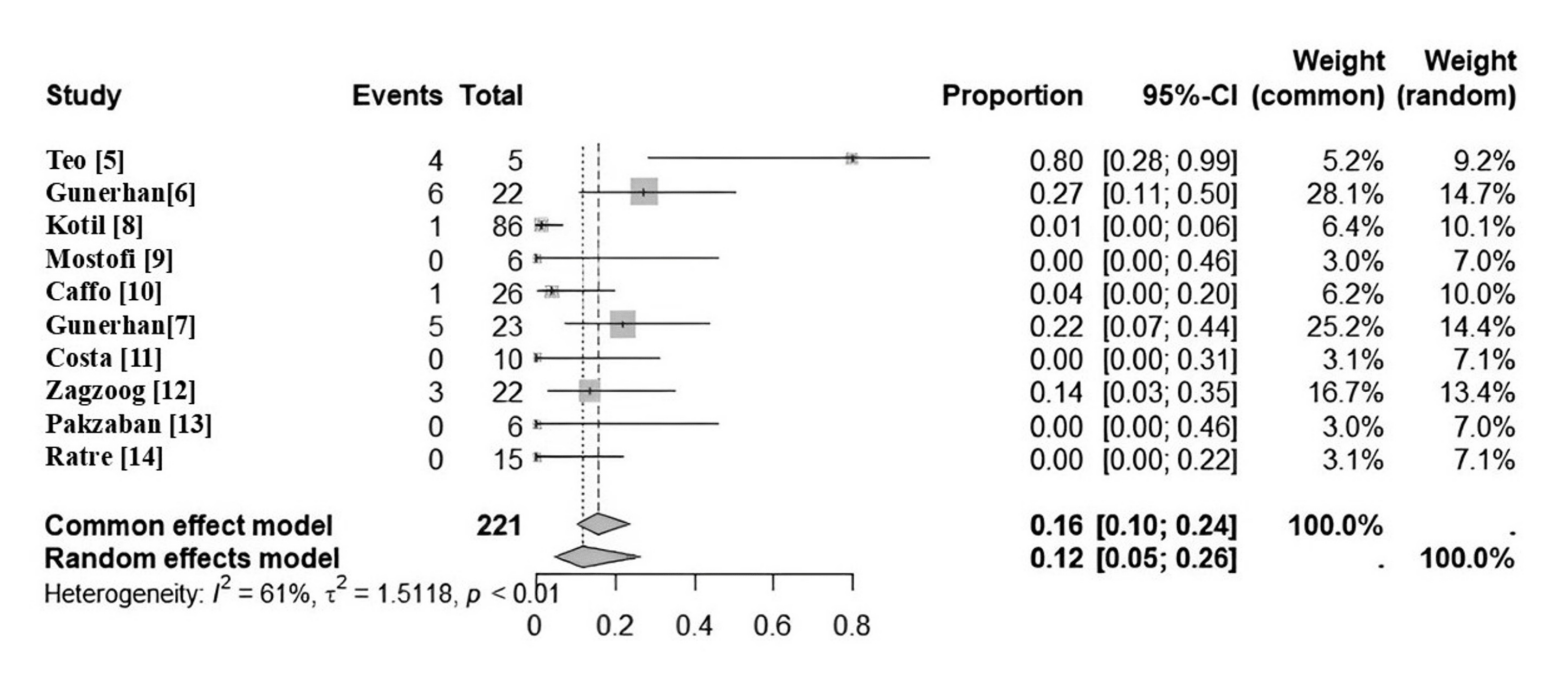
Forest plot detailing pooled complication rates and 95% confidence interval across all studies. I² = 61% shows moderate heterogeneity among complication rates. Source: Refs [5-14]

Out of 10 total, four surgeons reported no complications of any kind: Pazkaban et al. [13], Mostofi et al. [9], Costa et al. [11], and Ratre et al. [14]. Additionally, two surgeons reported only one complication of any kind: a single superficial wound infection and a single case of CSF leak, reported by Kotil et al. [8] and Caffo et al. [10], respectively. A meta-analysis of complication rates was conducted and is displayed in Figure 6. Moderate heterogeneity was observed (I² = 61%, t^2 ^= 1.5118, p < 0.01). A common effects model yielded an overall complication proportion of 0.16 (95% confidence interval: 0.1-0.24). A random effects model yielded a proportion of 0.12 (95% confidence interval: 0.05-0.26).

Discussion

This study was constructed with the intention of compiling peer-reviewed articles describing the minimally invasive techniques to treat CIM. The specificities of each minimally invasive method may vary with regard to retractor type, incision length, and more. With this fact in mind, our goal was to summarize the outcomes of minimally invasive Chiari decompression and to shed light on its effectiveness and complications.

Studies have shown that the MID foramen magnum decompression to treat CIM achieves equally favorable outcomes as the traditional open technique [19]. In addition, the literature has shown evidence of reduced complications from the MID in comparison to the traditional open technique. MID techniques require a smaller incision to be made, allowing for a greater chance of preserving skeletal muscle and ligaments needed to maintain biomechanics at the level of the craniocervical junction. This manifests as a reduced risk of neck pain/stiffness following the procedure. Furthermore, minimally invasive methods have been associated with reduced risk of bleeding and infection, as well as providing a faster recovery period after operation [5,18,19]. Upon review of the literature, MID techniques provide equal post-operative improvement in CIM patients when compared to the traditional open technique with reduced risk of post-operative complications.

Operation Time

Operation duration is another consideration when discussing the benefits of minimally invasive surgery. The meta-analysis of operative time yielded heterogeneous results, and the unique time of each surgeon had a high degree of variation. The wide range of operation times can be attributed to several factors, the first of which is the experience of the surgeon. Learning to use tubular retractors can be challenging for new surgeons [12]. Zagzoog et al. [12] claimed that it can take three to four surgeries for a novice surgeon to become comfortable with a minimally invasive technique. Thus, operations may take longer if the surgeon is less experienced with minimally invasive surgery. Second, the necessity of duraplasty per patient must be considered. While not all of the included studies mentioned which patients required duraplasty during the operation, four stated that a duraplasty was performed if necessary, a decision which was made in the operating room based on the surgeon’s direction. While the difference in mean operative times was not statistically significant (p = 0.12), it is worth noting that the mean duration for procedures with duraplasty was 33 minutes longer than the mean of the non-duraplasty group (115 minutes vs 82 minutes). Third, the variations of minimally invasive techniques also have to be taken into consideration. There are different types of minimally invasive techniques, such as tubular retractors, minimal incision with standard self-retaining retractors, and endoscopic. The intraoperative use of a microscope is also a factor.

Neurological Testing

Within the context of Chiari malformation repair, as well as in other neurosurgery, mJOA, KPS, and CCOS scores are used to monitor changes in patients’ living standards pre- and post-operatively. The mJOA assesses functional impairment experienced by patients as a complication of myelopathy by assessing scores of motor function in the upper and lower limbs, sensory innervation to the upper limb, and sphincter controls. It is graded on a scale from 0 to 18, with higher scores indicative of greater motor, sensory, and micturition control. Two papers within our systematic review utilized the mJOA, both of which were published by Günerhan et al. [6,7]. The first, a surgical technique paper recorded mJOA values of 14.5 ± 1.6/15.6 ± 1.0 pre- and post-operatively. The second, a comparative analysis of open surgery and neuroendoscopic approach recorded mJOA values of 14.5 ± 1.6/15.3 ± 1.3 pre- and post-operatively. The difference in mJOA values post-operatively via the minimally invasive procedure suggests improved motor, sensory, and micturition control.

Several studies within our systematic review utilized the KPS scaling, which is a measure of a patient's level of function. This test uses an 11-point scale ranging from fully functioning (100%) and death (0%). Two studies within our systematic review utilized KPS scoring, the first a neuroendoscopy-assisted study, in which Günerhan et al. [6] reported pre- and post-operative KPS scores of 81.8 ± 10.5/86.8 ± 6.5, respectively. The second is a prospective cohort study in which Ratre et al. [14] recorded average pre- and post-operative KPS scores of 78 and 93, respectively. The resultant KPS scores suggest an improvement in the functional status after a minimally invasive procedure.

In addition to using both mJOA and KPS scoring as a means of establishing post-operative success, two studies included within our systematic review opted to use the CCOS scaling. The CCOS assesses post-operative outcomes of Chiari improvement surgery based on scores of 1-4 given to four post-operative outcome categories: pain, nonpain symptoms, functionality, and complications (with a total of 16 points). Two papers utilizing CCOS scoring were published by Günerhan et al. [6,7]. Both studies by Günerhan et al. [6] described the number of patients who improved their CCOS post-operatively. The first one, a surgical technique paper, reported that 19 (86.3%) patients had an improved score, two (9.1%) had an unchanged score, and a single patient had an increased score. The second one, a comparative analysis of open surgery and a neuroendoscopic approach, saw that 20 (87%) patients improved their score post-operatively. Ratre et al. [14] also reported the CCOS of their patients; the average score was 15.5 with a range of 15-16. These scores indicate near or full improvement as per the 16-point scale. Thus, all three papers showed an average improvement in CCOS scores after minimally invasive surgery.

The VAS evaluates the changes in patients’ pain intensity and dizziness as it pertains to Chiari malformations; this metric scores pain on a scale of 1-10, with 0 indicating no pain. All studies included within our systematic review showed an improvement in the VAS post-operatively, suggesting that the minimally invasive procedure shows significant improvement in pain felt by the patient.

Syringomyelia 

CIM is one of the leading causes of syringomyelia [20]. Surgical intervention of CIM aims to reduce symptoms of that pathology. The effectiveness of surgery must be gauged at least in part by resolution of symptoms, many of which stem from syrinx development in the cervical spine. Thus, when considering techniques, syringomyelia improvement across all patients must be noted. In a systematic review and meta-analysis of traditional open foramen magnum decompression, Forander et al. [20] found that 65 (22%) of 298 surgeries without duraplasty and nine (26%) of 35 surgeries with duraplasty did not lead to syringomyelia improvement. Our review of minimally invasive surgery found that, across all reviews describing use of MID, 94 (84%) of 110 patients saw a reduction or improvement in syrinx volume; 16 (15%) saw no improvement. These results, while requiring further data and exploration, demonstrate that minimally invasive CIM decompression is as effective compared to the traditional approach for resolving syringomyelia among symptomatic patients.

Complications 

One of the main problems with foramen magnum decompression is the variability in the occurrence of complications; the rate of post-operative adverse events varies widely by institution and surgeon. In a systematic review of open foramen magnum decompression, Perrini et al. described the complication rate as ranging from 3%-40% [3]. With consideration given to these statistics, one goal of our analysis was to determine if minimally invasive surgery yielded similar variation. The highest rate of any study included was 40% (Teo et al. [5]), and the lowest rate achieved was 0% (Pakzaban et al. [13] and Ratre et al. [14]). The meta-analysis demonstrated moderate heterogeneity, indicating that the rate of complications still varies to some degree per surgeon and institution, regardless of whether the technique used is open or minimally invasive.

Our statistical analysis of complication rates is notable. In a systematic review of open surgery for CIM, Zhao et al. [1] found that complications (adverse events) occurred in 242 out of 1,242 patients (19.6%). While the available sample of patients who underwent MID is smaller, the proportion of patients who experienced complications is lower (11%). Excluding outlier data (Teo et al. [5]), the complication rate becomes even more favorable at 7.5%. Meta-analysis of complication rates confirms that those proportions are statistically significant. Thus, our study can affirm the idea that minimally invasive surgery results in lower complication rates. Since minimally invasive surgery by definition exposes less tissue to pathogens due to its small incision area, we expected to see a low occurrence of post-operative infection. Indeed, our review yielded only a single occurrence of superficial wound infection out of 200 patients.

Minimally invasive surgery may offer additional benefits, such as reduced scarring and potentially decreased blood loss. While blood loss during surgery is hard to quantify and often relies on visual estimate, smaller incisions and surgical areas are conducive to lower amounts of blood loss during many similar operations [20]. Thus, the use of many other minimally invasive techniques is supported by surgeons who believe that such operations may result in less blood loss, scarring, infection, and shorter hospital stay [3], and minimally invasive Chiari decompression is no exception.

Limitations

The small volume of existing literature surrounding MID contributes to certain limitations of this study. First, this review included a relatively small overall sample size. With only 200 patients undergoing MID across the included studies, the generalizability of the conclusions is limited. Additionally, considerable heterogeneity exists across the included studies in terms of surgical technique, including variations in incision size, the use of duraplasty, retractor types, and adjunct tools such as endoscopes and microscopes. This diversity complicates direct comparisons and weakens the consistency of outcome interpretation, highlighting the need for further exploration of which operative factors affect surgical outcomes. Furthermore, only a subset of studies included comparison groups undergoing open surgery, reducing the strength of head-to-head outcome analysis. In addition, many of the included studies were observational or case series in design, introducing the potential for selection and publication bias.

In most studies, reporting of comorbidities was extremely limited, only including a discussion of presenting symptoms of the patient cohort. Additionally, which patients experienced each particular symptom was not explicitly described in several cases. This inconsistency in comorbidity and symptomatic reporting highlights the need for further literature to describe such confounders distinctly.

There was also variability in outcome measurement, as not all studies employed standardized clinical scoring systems such as the mJOA, KPS, CCOS, or VAS, and even among those that did, reporting was inconsistent. Finally, the learning curve associated with MID was not uniformly addressed, as surgeon experience may influence both operative time and complication rates. These limitations collectively highlight the need for larger, prospective, multicenter studies with standardized protocols and longer-term follow-up to further evaluate the role of MID in the treatment of CIM. Expansion of the current literature surrounding MID will enable further studies to address these challenges.

## Conclusions

Chiari malformation decompression is a procedure that incurs the possibility of many different complications. As with any invasive procedure, the search for safer, more efficient, and patient-specific techniques is one that is ongoing and relevant to the field of neurological surgery. CIM is no exception, with anywhere from 30% to 50% of patients experiencing symptoms related to that condition. All symptomatic patients are offered surgical intervention, as foramen magnum decompression results in significant symptomatic improvement. While traditional open decompression is still the mainstay of treatment, the minimally invasive techniques have become more prominent; the goals of endoscopic or other minimally invasive techniques are to reduce complications and improve patient outcomes. Across several types of neurological and functional testing (KPS, VAS, CCOS, etc.), patients who underwent minimally invasive decompression showed excellent improvement in function and syrinx-related symptoms. These results demonstrate that this technique is effective for the treatment of symptomatic CIM. While the rates of all other complications varied, meta-analysis found that their proportions were lower on average than open surgery. These findings indicate that minimally invasive decompression is a promising alternative to open surgery. While further research into this procedure is warranted, minimally invasive foramen magnum decompression produces compelling results, and neurosurgical institutions and surgeons alike would benefit from its study and practice.
